# The need for clinical, genetic and radiological characterization of atypical polycystic kidney disease

**DOI:** 10.1007/s40620-024-02181-6

**Published:** 2025-02-10

**Authors:** Matteo Righini, Cristiana Corsi, Nicola Sciascia, Valeria Aiello, Francesca Ciurli, Sarah Lerario, Gian Marco Berti, Francesca Montanari, Amalia Conti, Carlotta Pia Cristalli, Soara Menabò, Luca Caramanna, Francesco Tondolo, Daniela Turchetti, Gaetano La Manna, Irene Capelli

**Affiliations:** 1 Nephrology and Dialysis Unit, Santa Maria Delle Croci Hospital, Ravenna, Italy; 2 Department of Electrical, Electronic and Information Engineering “Guglielmo Marconi”, University of Bologna, Bologna, Italy; 3 Radiology Unit, IRCCS Azienda Ospedaliero-Universitaria Di Bologna, Bologna, Italy; 4 Nephrology, Dialysis and Kidney Transplant Unit, IRCCS Azienda Ospedaliero-Universitaria Di Bologna, Bologna, Italy; 5 Department of Medical and Surgical Sciences (DIMEC), Alma Mater Studiorum, University of Bologna, Bologna, Italy; 6 Medical Genetics Unit, IRCCS Azienda Ospedaliero-Universitaria Di Bologna, Bologna, Italy

**Keywords:** ADPKD, Atypical, Total kidney volume, Genetics

## Abstract

**Background:**

Autosomal Dominant Polycystic Kidney Disease (ADPKD) is a monogenic disease having a prevalence of 1:400–1000 live births. Depending on kidney imaging, patients can be subdivided into Class 1 (typical) and Class 2 (atypical). The present study aims to provide better assessment of Class 2 patients to help define their family history, together with their clinical and radiological characteristics.

**Methods:**

One hundred twenty-four PKD patients with abdominal Magnetic Resonance Imaging (MRI) for the staging of ADPKD, were retrospectively analyzed, aiming to focus on Class 2 ADPKD patients. Total kidney volume and total cyst volume were evaluated, while also assessing their clinical and genetic characteristics.

**Results:**

Twelve patients fulfilled the Mayo criteria for Class 2 ADPKD (two Class 2B and ten Class 2A). Extrarenal involvement was observed in 66.7% of cases, but only two subjects presented an estimated Glomerular Filtration Rate (eGFR) < 60 mL/min/1.73 m^2^. A positive family history for cystic disease was more frequent compared to other published cohorts. Only 8.3% tested positive for a likely pathogenic mutation in the *PKD*1 gene. Class 2B patients showed a lower height-adjusted total kidney volume, with a lower percentage of total cyst volume.

**Conclusion:**

Based on our results, atypical ADPKD does not represent an uncommon condition, being present in about 10% of MRI-evaluated patients diagnosed with ADPKD. Genetic tests are frequently negative for *PKD1/PKD2*, and total cyst volume and residual tissue volume do not increase the prognostic value of MRI in patients with these radiological characteristics. Other tools are needed to better characterize their kidney prognosis.

**Graphical abstract:**

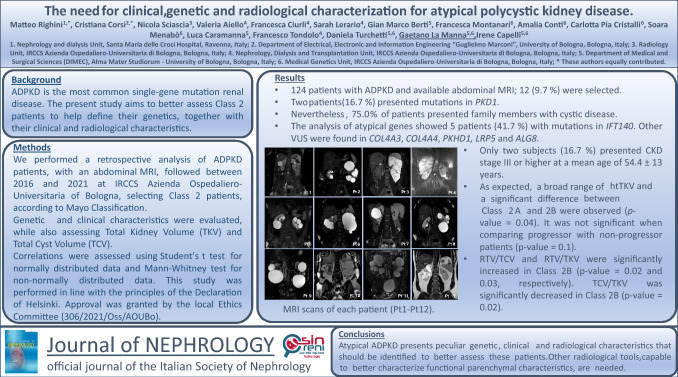

## Introduction

Autosomal Dominant Polycystic Kidney Disease (ADPKD) is one of the most common life-threatening monogenic disorders, presenting a worldwide prevalence of 1:400–1000 live births. About 50% of adult ADPKD patients will require dialysis or kidney transplantation within their 6th decade of life [1]. Clinical management of patients with ADPKD is challenging: its presentation varies significantly between and within affected families, and it progresses for several decades before reaching kidney failure. Major factors identified as disease progression predictors include genotype, sex, age, urological events, hypertension, and total kidney volume. These features were used to develop two main scores: the Predicting Renal Outcome in Polycystic Kidney Disease (PROPKD) score and the Mayo Clinic Imaging Classification (MCIC) [2]. The latter is based on kidney imaging: patients with bilateral diffuse cystic involvement throughout the kidney parenchyma and no kidney atrophy are classified as Class 1 (typical), defining the majority of ADPKD patients (95%), while a minority of patients (5%) are classified as Class 2 (atypical). Regarding Class 1, patients are divided into five sub-classes (1A-1E) depending on the total kidney volume. Indeed, the total kidney volume is associated with estimated Glomerular Filtration Rate (eGFR) decline and, therefore, patients defined as rapid progressors are assigned to classes 1C-1D-1E, i.e., with higher total kidney volume [3]. Total kidney volume is recognized as the most valuable predictor of ADPKD progression, and is widely used as an end point in clinical trials [4, 5]. Patients in Class 2 are further divided into Class 2A if they present focal disease (unilateral, segmental, asymmetric or bilateral atypical presentation), and Class 2B in case of atrophic disease (bilateral or unilateral acquired atrophy) [3]. Despite the useful model, total kidney volume in Class 2 patients is not directly linked to eGFR decline, thus predicting the risk of progression in these patients is an issue that remains unresolved [3, 6]. Moreover, extrarenal involvement and familiality are also poorly described, as are the genetic aspects. Bae et al*.* [5] carried out a clinical study to predict disease progression in Class 2 patients, reclassifying Class 1 patients with exophytic cysts and allowing Class 2 patients to be included in the Mayo classification. This increased the specificity of total kidney volume in predicting the development of Chronic Kidney Disease (CKD) stage III in all participants [5].

The aim of the present study was to better characterize the clinical presentation of Class 2 patients in order to predict disease progression ideally with the same reliability as Class 1 patients.

## Methods

### Imaging data acquisition

All ADPKD patients followed in our out-patient clinic between 2016 and 2021 who had undergone abdominal Magnetic Resonance Imaging (MRI) for ADPKD staging were retrospectively analyzed, according to the unified ultrasonographic criteria [7]. MRI study was carried out on a General Electric 1.5 T through Sagittal T1 Fast Spin Echo (FSE), Axial Fluid Attenuated Inversion Recovery (FLAIR), Coronal T2 FSE, Diffusion Weighted Imaging (DWI), Axial T2.

Class 2 ADPKD patients were selected from among our ADPKD population, based on the MCIC [3]. Two experienced radiologists from two different institutions, blinded to the clinical data, independently reviewed the images. The two radiologists determined both the single kidney and the total kidney volume, and then matched the data assigning each patient to his/her specific Mayo class. Disagreements were resolved by a third highly experienced radiologist. Nephromegaly was defined as total kidney volume > 750 ml [8].

Total cyst volume was automatically assessed by studying the distribution of gray level intensities within the kidney. A progressive weighted curve (PWC) was defined as [9]:

$$PWC=\frac{{\sum }_{i=0}^{k}{w}_{i}{x}_{1}}{{\sum }_{i=0}^{k}{w}_{i}}$$

where *w*_*i*_ represents the histogram count of the i^th^ gray level and *x*_*i*_ represents the corresponding gray intensity value. For each value *k*, the progressive weighted curve value represents the weighted average of the intensity values up to the *k*^th^ gray level, in which the histogram counts define the weights. The inflection points of the progressive weighted curve correspond to the candidate thresholds for kidney region segmentation. The selection among these thresholds was performed by assigning a score to each candidate threshold. The score was computed by considering a set of features extracted from the objects resulting from kidney region segmentation. Each object detected by applying a candidate threshold was characterized by its shape in terms of circularity, standard deviation of the gray level intensity within it and its mean border intensity gradient; this score was then used to define the best threshold for cyst segmentation.

Total cyst volume was then subtracted from total kidney volume, while the remaining part of the vital kidney tissue was defined as residual tissue volume. TCV, TKV and RTV were evaluated in progressor (eGFR slope > 1 mL/min/1.73 m^2^/year) and non-progressor patients.

### Clinical information

Demographic information (sex and age), kidney function parameters (creatinine values, eGFR values, eGFR slope, stage of CKD), clinical parameters (presence/absence of arterial hypertension and urological events, use of Angiotensin Converting Enzyme (ACE) inhibitors, family history of ADPKD or family history of hemorrhagic stroke and/or subarachnoid hemorrhage, smoking habit) and the results of genetic tests were collected from the clinical charts. All parameters were collected at the time of MRI image acquisition. Family history was considered positive in case of a diagnosis of ADPKD in a first degree relative.

### Genetic testing

Targeted Next Generation Sequencing on *PKD1* (MIM#601,313 HGNC:9008, RefSeq NM _001009944.3) and *PKD2* (MIM#173,910 HGNC:9009, RefSeq NM_000297.4) genes was performed in our laboratory using genomic DNA isolated from ethylenediaminetetraacetic acid (EDTA) peripheral blood by the semi-automatic Maxwell 16 instrument (Promega Corporation, Madison, WI USA) [10,) (11]. When *PKD1* and *PKD2* sequencing and multiplex ligation-dependent probe amplification (MLPA) analysis tested negative, we performed targeted next generation sequencing using a panel of 18 candidate atypical cystic genes, as reported below: *GANAB_NM_198335.4, DZIP1L_NM_173543.3, DNAJB11_NM_016306.5, ALG9_NM_001077690.1, PRKCSH_NM_002743.3, SEC63_NM_007214.5, ALG8_NM_024079.5, LRP5_NM_002335.4, SEC61B_NM_006808.3, IFT140_NM_014714.4, HNF1B_NM_000458.4, UMOD_NM_001008389.3, REN_NM_000537.4, SEC61A1_NM_013336.4, BICC1_NM_001080512.3. PKHD1_NM_138694.4, COL4A3_ NM_000091.5*, *COL4A4_ NM_000092.5,* and *COL4A5_ NM_033380.3*. Additionally, all patients underwent targeted next generation sequencing using a panel including the following list of genes related to several genetic kidney conditions: *NPHP1_NM_001128178.3, NPHP3_NM_153240.5, NPHP4_NM_015102.5, NPHP5_NM_001023570.4, NPHS1_NM_004646.4, NPHS2_NM_014625.4, TSC1_NM_000368.5, TSC2_NM_000548.5, ANLN_NM_018685.5, CRB2_NM_173689.7, CLCNKA_NM_004070.4, CLCNKB_NM_000085.5, DGKE_NM_003647.3, DSTYK_NM_015375.3, INF2_NM_022489.4, MYO1E_NM_004998.4, PAX2_NM_000278.5, PLCE1_NM_016341.4, PTPRO_NM_030667.3, SEC63_NM_007214.5, SLC12A3_NM_001126108.2, TBX18_NM_001080508.3, TRPC6_NM_004621.6, WT1_NM_024426.6.*

Variants were classified in accordance with the American College of Medical Genetics and Genomics (ACMG) guidelines listing specific standard terminology: “P” for pathogenic (ACMG 5), “LP” for likely pathogenic (ACMG 4), “VUS” for variant of uncertain significance (ACMG 3), “LB” for likely benign (ACMG 2), and “B” for benign (ACMG 1) [12]. Genetic counseling was provided before and after genetic testing, according to clinical practice.

### Statistical analysis

We presented continuous data as median, mean ± Standard Deviation. Correlations were assessed using Pearson’s or Spearman’s method, for normally or non-normally distributed data, respectively. We used Student t test for normally distributed data and Kruskal-Wallis test for non-normally distributed data. A p-value < 0.05 was considered significant.

## Results

### Patients’ overview

**Fig. 1:**
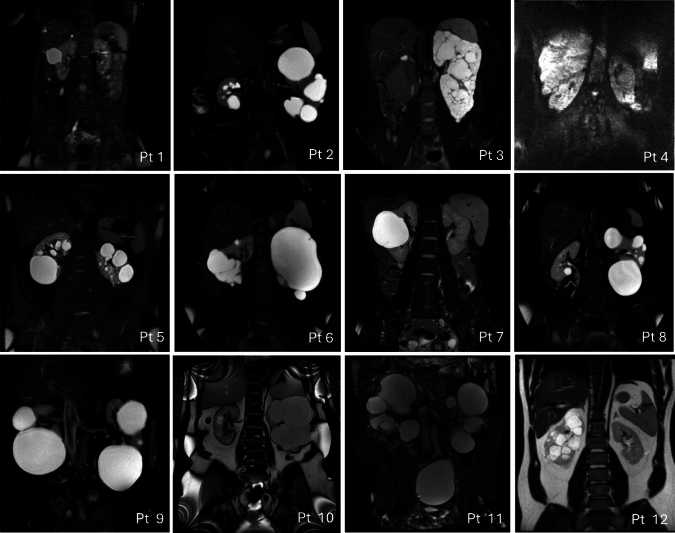
MRI scans of each patient (Pt1-Pt12). (Pt1) Class 2B, bilateral atrophy presentation; (Pt2) Class 2A, lopsided presentation; (Pt3) Class 2A, unilateral presentation; (Pt4) Class 2B, bilateral atrophy presentation; (Pt5) Class 2A, lopsided presentation; (Pt6) Class 2A, lopsided presentation; (Pt7) Class 2A, mild lopsided presentation; (Pt8) Class 2A, asymmetric presentation; (Pt9) Class 2A, lopsided presentation; (Pt10) Class 2A, asymmetric presentation; (Pt11) Class 2A, lopsided presentation; (Pt12) Class 2A, unilateral presentation

During the study period, 221 ADPKD patients were evaluated. Among them, 124 underwent abdominal MRI, and 12 patients (9.7%) fulfilled the Mayo criteria for Class 2 ADPKD [6]. Each patient belonged to a different family. Images highlighting the radiological involvement of each patient are shown in Fig. 1, the clinical features are shown in Table 1, and the genetic characteristics in Table 2. Each patient was assigned a number that remained the same in all Tables and Figures. The initial nephrology referral, that allowed the specific diagnosis of atypical ADPKD, followed an incidental finding of kidney cysts in most cases (41.7%). The presence of a positive family history (occurring in 33.3% of cases), or symptoms (in 25%), on the other hand, were less likely to be the main indication for referral even though both played a relevant role. Mean age at referral was 54.4 ± 13 years, mean serum creatinine was 0.97 ± 0.25 mg/dL, and mean eGFR was 74.2 ± 15.9 mL/min/1.73 m^2^. Only two subjects (16.7%) presented an eGFR < 60 mL/min/1.73 m^2^. The majority of patients presented hypertension (58.3%), and 33.3% were on treatment with ACE inhibitors. Among patients who presented extrarenal involvement (66.7%), six (50.0%) had liver cysts, three (25.0%) had intracranial aneurysms, one (8.3%) had mitral valvulopathy, one (8.3%) had diverticulitis, and none had pancreatic cysts. Nine (75.0%) patients had a positive family history for cystic kidney disease.

**Table 1:** Clinical features of atypical ADPKD patients in our cohort

Patient	Age [years]	Sex [M/F]	Serum Creatinine [mg/dL]	eGFR [mL/min/1.73 m^2^]	Annual eGFR slope [mL/min/1.73 m^2^/year]	Hypertension [Y/N]	Initial indication for nephrology referral	Smoking [Y/N]	Diabetes [Y/N]	Extra-renal involvement [Y/N]
Pt1	44	F	0.7	100	0.78	N	US for FH	N	N	Y
Pt2	68	F	0.96	61	-0.86	Y	Incidental finding	N	Y	N
Pt3	32	F	0.82	88	-1.1	N	US for symptoms	N	N	N
Pt4	47	F	0.76	86	1.22	Y	US for FH	Former	N	Y
Pt5	48	M	1.4	57	8.59	Y	Incidental finding	N	N	N
Pt6	49	F	0.71	72	0.83	Y	US for symptoms	N	N	Y
Pt7	45	F	0.8	77	-2.23	N	US for FH	N	N	N
Pt8	63	F	0.76	84	-3.35	N	US for FH	N	N	Y
Pt9	71	M	1.13	78	-1.96	Y	Incidental finding	N	N	Y
Pt10	69	F	1.27	43	-7.14	Y	Incidental finding	N	N	Y
Pt11	70	M	1.2	62	-0.92	Y	US for symptoms	Y	N	Y
Pt12	47	M	1.14	82	1.36	N	Incidental finding	N	N	Y

*Pt* patient, *M* male, *F* female, *eGFR* estimated glomerular filtration rate, *Y* yes, *N* no, *US* ultrasound, *FH* family history

**Table 2:** Genetic characteristics of atypical ADPKD patients in our cohort

Patient	Family history	Gene	Genetics
Pt1	Yes Mother (70 y) with PKD (positive for FM); maternal grandmother HD KRT (60 y), now deceased, not tested for FM; daughter (16 y) WT for FM; son (13 y) WT for FM	PKD1	NM_001009944.3: c.2180 T > C p.Leu727Pro [ACMG 4]
Pt2	Yes Father with PKD and AMI, died of AMI at 69 y, not tested for FM; brother with PKD, died of AMI at 65 y, not tested for FM; sister (59 y) US negative for cysts, not tested for FM; son (45 y) US negative for cysts, not tested for FM; son (48 y) US negative for cysts, not tested for FM	COL4A4	NM_000092.5: c.2860 + 3A > C [ACMG 3]
Pt3	No	PKHD1	NM_138694.4: c.5134G > A p.Gly1712Arg [ACMG 3]
Pt4	Yes Father (72 y) nephrectomy (50 y), HD KRT (65 y), CV disease, not tested for FM; paternal grandmother affected by PKD, not tested for FM; paternal uncle with PKD, not tested for FM; brother (52 y) US negative for cysts, not tested for FM	/	WT
Pt5	Yes Sister (57 y) WT for FM; brother (60 y) positive for FM; brother (65 y) positive for FM	/	WT
Pt6	Yes Mother with HT, not tested for FM; sister (56 y) with kidney cysts and ICA, WT for FM	IFT140^a^ COL4A3^b^	^a^NM_014714.4: c.1863_1866del p.Glu623ArgfsTer20 [ACMG 5] ^b^NM_000091.5: c.4421 T > C p.Leu1474Pro [ACMG 3]
Pt7	Yes Mother (80 y) with PKD, KRT (55 y), positive for FM; maternal uncle (76 y) with PKD, not tested for FM	IFT140^a^ PKD1^b^ ALG8^c^	^a^NM_014714.4: c.2766_2768 + 1del [ACMG 4] ^b^NM_001009944.3: c.194 T > A p.Ile65Asn [ACMG 3] ^c^NM_024079.5: c.980C > G p.Thr327Arg [ACMG 3]
Pt8	Yes Mother with PKD, deceased at 90 y, not tested for FM; brother (62 y) with PKD, not tested for FM; maternal uncle with PKD, deceased at 80 y, not tested for FM; 3 sons without cysts cysts at US, not tested for FM	IFT140^a^ ALG8^b^	^a^NM_014714.4: c.919C > T p.Arg307Ter [ACMG 5] ^a^NM_014714.4: c.2797G > A p.Glu933Lys [ACMG 3] ^b^NM_024079.5: c.980C > G p.Thr327Arg [ACMG 3]
Pt9	No	IFT140	NM_014714.4: c.1501C > T p.Arg501Ter [ACMG 5]
Pt10	No	IFT140	NM_014714.4: c.1501C > T p.Arg501Ter [ACMG 5]
Pt11	Yes Mother died at 81 y of cancer, with kidney cysts, not tested for FM; father died at 49 y of AMI, not tested for FM; brother with kidney cysts, not tested for FM	LRP5	NM_002335.4: c.3107G > A p.Arg1036Gln [ACMG 3]
Pt12	Yes Father (65 y) with kidney cysts, not tested for FM; 2 brothers without cysts at US, not tested for FM	/	WT

In the Genetics column, variants are expressed with their characteristics: HGVS classification; ACMG nomenclature

*Pt* patient, *WT* wild type, *y* years, *FM* family mutation, *HD* hemodialysis, *KRT* kidney replacement therapy (dialysis/transplant), *AMI* acute myocardial infarction, *US* ultrasound, *CV* cardiovascular, *HT* hypertension, *ICA* intracranial aneurysm

### Genetic results

Concerning genetic aspects, *PKD1* variants were found in two patients (16.7%): one (8.3%; Patient 1) carried a likely pathogenic variant (c.2180 T > C_p.Leu727Pro, exon 11, classified as Likely Pathogenic in ClinVar and Varsome Premium databases, CADD 23.5) and one (8.3%; Patient 7) carried a variant of uncertain significance (c.194 T > A p.Ile65Asn). *PKD2* variants were absent in the analyzed cohort, although atypical ADPKD-associated genes were found in several cases. The most relevant was *IFT140*, found to be mutated in 5 patients (41.7%). Patient 8 presented two different variants, one pathogenic and one variant of uncertain significance of the gene (c.919C > T p.Arg307Ter and c.2797G > A p.Glu933Lys). Patient 6 (c.1863_1866del p.Glu623ArgfsTer20), Patient 9 (c.1501C > T p.Arg501Ter) and Patient 10 (c.1501C > T p.Arg501Ter) carried *IFT140* pathogenic variants (P; ACMG 5), while Patient 7 presented a likely pathogenic *IFT140* variant (c.2766_2768 + 1del; ACMG 4) (Table 2). Other mutated genes observed in our cohort were *COL4A4*, *COL4A3*, *PKHD1, LRP5* and *ALG8*. Patient 2 carried a variant of uncertain significance in the *COL4A4* gene (c.2860 + 3A > C, intron 31, classified as a variant of uncertain significance in ClinVar and Varsome Premium databases, CADD 17.13), Patient 3 had a variant of uncertain significance in *PKHD1* (c.5134G > A p.Gly1712Arg), Patient 6 showed a variant of uncertain significance in *COL4A3* (c.4421 T > C p.Leu1474Pro), while Patients 7 and 8 both carried the same variant of uncertain significance in *ALG8* (c.980C > G p.Thr327Arg). Patient 7 hence presented two variants of uncertain significance, in *PKD1* and in *ALG8*. Finally, Patient 11 carried a variant of uncertain significance in *LRP5* (c.3107G > A p.Arg1036Gln).

### Imaging results

**Fig. 2:**
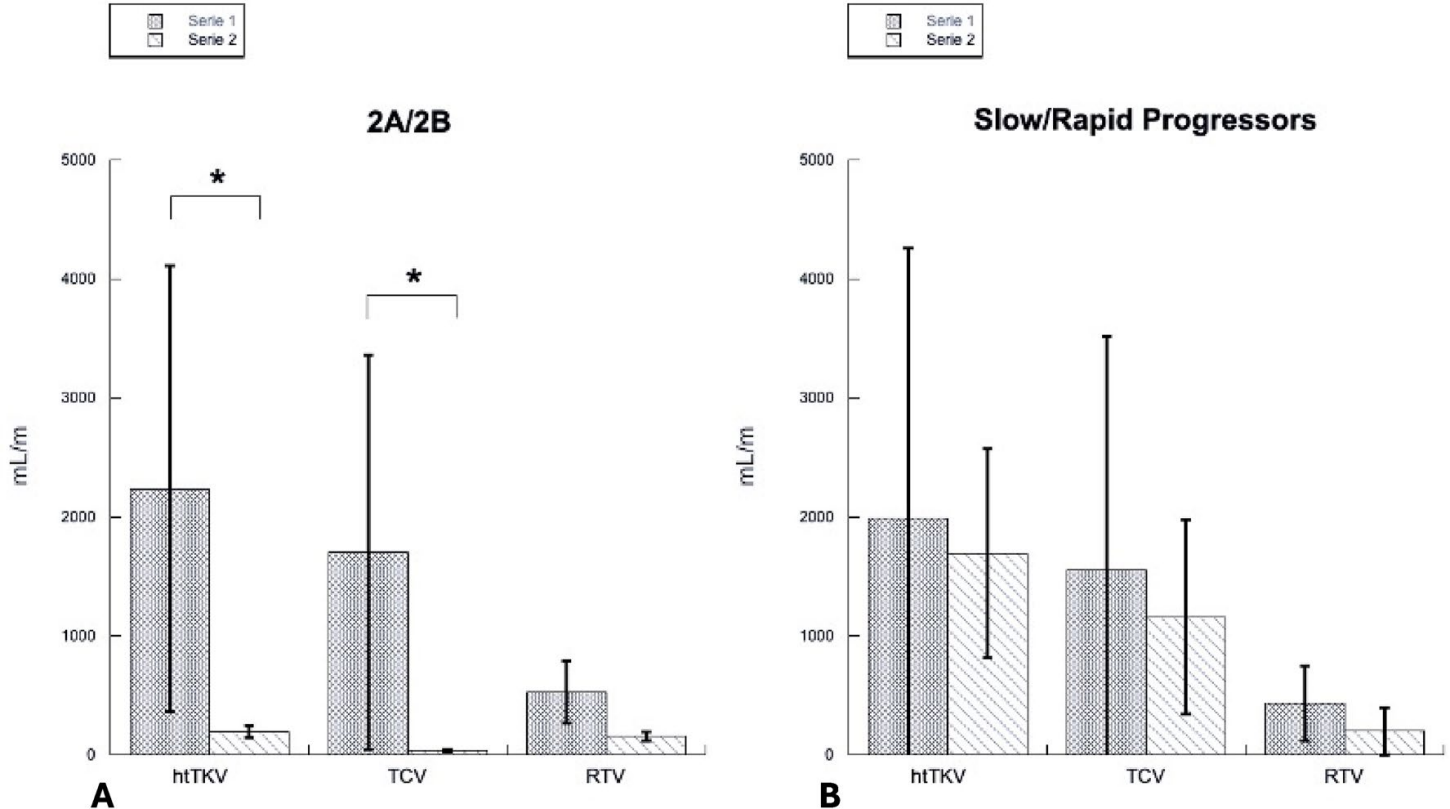
**A** Comparison between Class 2A and 2B patients’ htTKV, TCV and RTV. *p value < 0.05. **B** Comparison of htTKV, TCV and RTV between progressors and non-progressors

With regard to the MRI evaluation, the patients’ characteristics are described in Table 3. According to the classification presented by Iliuta et al. [13], Class 2 patients from our cohort presented different phenotypes: two subjects were Class 2B with bilateral atrophy, while the other ten patients were Class 2A, two of whom showed asymmetric presentation, two unilateral, five lopsided and one mild lopsided. As expected, a broad range of height-adjusted total kidney volumes were observed, with a mean value of 1893.3 ± 1871.6 mL/m. Furthermore, the difference between height-adjusted total kidney volume in Class 2A and 2B patients was assessed, although the significance was foreseen by the very definition of Class 2A and 2B radiological characteristics (Fig. 2). Class 2A patients presented a mean height-adjusted total kidney volume of 2232.2 ± 1874.9 mL/m, while in Class 2B patients it was 198.5 ± 50.2 mL/m (*p* value = 0.04).

**Table 3:** MRI data of atypical ADPKD patients

Patient	Type	htTKV [mL/m]	TCV [mL/m]	Percentage [%]	RTV [mL/m]
Pt1	Bilateral atrophy	163	28	17.5	135
Pt2	Lopsided	1644	1216	73.9	428
Pt3	Unilateral	1630	1313	80.5	317
Pt4	Bilateral atrophy	234	47	20.3	187
Pt5	Lopsided	574	311	54.1	263
Pt6	Lopsided	5571	4663	84.9	908
Pt7	Mild lopsided	581	230	39.6	351
Pt8	Asymmetric	1472	914	62.1	558
Pt9	Lopsided	2620	2201	84	419
Pt10	Asymmetric	2100	1295	61.7	805
Pt11	Lopsided	5532	4600	83.1	932
Pt12	Unilateral	598	293	49	305
		1893.3 ± 1871.6	1425.9 ± 1632.1	59.2 ± 23.9	467.3 ± 274.6

Types of atypical ADPKD patients listed in the second column, described according to the Iliuta et al. (13) classification

*Pt* patient, *htTKV* height-adjusted total kidney volume, *TCV* total cyst volume, *RTV* residual tissue volume

We also used total cyst volume and residual tissue volume to better define Class 2 patients. In Class 2B patients, the total cyst volume was lower (37.5 ± 13.4 mL/m) compared to Class 2A patients (1703.6 ± 1655.8 mL/m), with a mean lower percentage of total cyst volume (2A: 67.3% vs 2B: 18.9%) (*p* value = 0.01). Patients with unilateral involvement presented a greater total cyst volume. Concerning residual tissue volume, the mean value was 467.3 ± 274.6 mL/m, including six patients presenting residual tissue volume < 400 mL/m, although only one had impaired kidney function. Residual tissue volume in Class 2A was 528.6 ± 258.8 mL/m, while it was 161 ± 36.8 mL/m in Class 2B (*p* value = 0.2). Residual tissue volume/total cyst volume was significantly higher in Class 2B (4.40 ± 0.60) compared to Class 2A (0.58 ± 0.45) (*p* value = 0.02).Residual tissue volume/total kidney volume was also significantly higher in Class 2B (0.81 ± 0.02) compared to Class 2A (0.33 ± 0.16) (*p* value = 0.03). Lastly, total cyst volume/total kidney volume was significantly higher in Class 2A (0.67 ± 0.16) compared to Class 2B (0.19 ± 0.02) (*p* value = 0.02).

### Disease progression

Finally, we calculated the annual slope of the eGFR for each patient, identifying four subjects with a significant decline in eGFR slope (progressors). The median eGFR slope was -0.89(-2.03–0.93) mL/min/1.73m^2^/year. We then compared the htTKV, TCV and RTV of the four progressors with those of the non-progressors (Fig. 2). The height-adjusted total kidney volume was 1786.00 (1249.25–2230.00) mL/m vs 1114.00 (489.00–2616.00) mL/m (*p* value = 0.610), while the TCV was 1104.50 (743.00–1521.50) mL/m vs 763.50 (231.50–2134.75) mL/m (*p* value = 0.865). The RTV was 488.50 (402.00–619.75) mL/m in progressors and 311.00 (244.00–548.00) mL/m in non-progressors, with a p-value of 0.308. RTV/htTCV was lower in non-progressors (0.60(0.23–1.78)) compared to progressors (0.62(0.51–0.85)) (*p* value = 0.734). RTV/htTKV was also lower in non-progressors (0.36 (0.19–0.58)) compared to progressors (0.38(0.32–0.44)) (*p* value = 0.734). Lastly, TCV/htTKV was lower in progressors (0.62(0.56–0.68)) compared to non-progressors (0.64(0.42–0.81)) (*p* value = 0.734).

## Discussion

Most ADPKD patients (estimated as 95%) present with bilateral and diffuse distribution of the disease, while a minority have an atypical imaging pattern (Class 2 of Mayo Clinic Imaging Classification) [6]. Despite the achievements in the management of ADPKD patients, there are currently no strong predictors for disease progression in Class 2 ADPKD, mainly because of the lack of large population studies, which is also why the prevalence of this specific population is still not fully known. The incidence of Class 2 ADPKD patients in our cohort was consistent with other single-center published cohorts (5.4% vs 5–10%) [5]. A comparison of the patients’ characteristics between the Bologna cohort and the cohorts described in the literature can be found in Table 4.

**Table 4:** Comparison of clinical features in atypical ADPKD patients from our cohort and from cohorts described in the literature [3, 6, 13]

	Irazabal et al. 2015 CRISP (173 pts)	Irazabal et al. 2015 MTPC (590 pts)	Irazabal et al. 2017 HALT-PKD (551 pts)	Iliuta et al. 2023 (523 pts)	Bologna cohort 2024 (124 pts)
Class 2 patients [n°]	4	52	30	46	12
Prevalence (%)	2.3	8.8	5.4	8.8	9.7
Age [mean (range)] or [mean ± SD] (or Q1-Q3)	30 (25–46)	61 (50–71)	41.9 ± 5.6	55 (45–68)	54.4 ± 13
Females [%]	50.0	46.2	70.0	37.0	66.7
Family History [%]	75.0	48.1	N/A	26.1	75.0
Genetic analysis [%]	100.0	25.0	93.3	100.0	100.0
NMD [%]	25.0	38.5	14.3	80.4	33.3
PKD1 [%]	50.0	61.5	46.4	8.7	16.7
PKD2 [%]	50.0	0.0	39.3	10.9	0.0
htTKV (mL/m) [median (Q1–Q3)] or [mean ± SD]	2A: 886.0(754.0–1574.0) 2B: 260.0	2A: 591.0(476.0–1020.0) 2B: 351.0(248.0–481.0)	818.2 ± 518.2	634.0(457.0–874.0)	1551.00(579.2–2230.0)
eGFR (mL/min/1.73 m^2^) [median (Q1–Q3)] or [mean ± SD]	2A: 52.0(51.0–55.0) 2B: 74.0	2A: 67.0(57.0–80.0) 2B: 33.0(21.0–50.0)	90.3 ± 14.8	82.0(68.8–98.5)	72.5(58.5–81.8)
Hypertension [%]	N/A	N/A	N/A	N/A	58.3
Extrarenal involvement [%]	N/A	N/A	N/A	87.0	66.7

*F* females, *NMD* no mutation detected, *htTKV* height-adjusted total kidney volume, *eGFR* estimated glomerular filtration rate. *N*/*A* not assessed

 Class 2 ADPKD patients need to be better characterized with regard to family history, genetics, and clinical features, like kidney function decline, urological events, hypertension, and extrarenal manifestations. As for family history, 75.0% of our population presented a positive family history, similar to what was reported for Class 1 patients [13]. These data aligned with some other reported cohorts [3,) (14,) (15], while in the Iliuta et al. [13] cohort, the prevalence of a positive family history was extremely low [3]. We evaluated all of our patients’ family members who were willing to undergo genetic testing and sonography to detect the presence of cystic disease. Many family members of our cohort had never been genetically tested, highlighting the possibility of missed diagnosis in cases of cystic kidney diseases regardless of progression to kidney failure. We believe that the high percentage of positive family history we found is indicative of the need for nephrological and genetic assessment of all family members of patients affected by  this specific phenotype.

Among our cohort, 83.3% did not carry any mutation in the *PKD1* or *PKD2* genes. Two patients (16.7%) carried a *PKD1* mutated gene: one subject had a non-truncating variant classified as likely pathogenic (NM_001009944.3: c.2180 T > C p. Leu727Pro), while the second one presented a variant of uncertain significance (NM_001009944.3: c.194 T > A p.Ile65Asn). In the study conducted by Iliuta et al. [14], 30.4% of Class 2 ADPKD patients presented a truncating *PKD1* mutation, 30.4% had a *PKD2* mutation and 39.2% did not present any variant. In our study, the percentage of typical mutated genes was much lower (16.7%). Thus, we decided to search for the involvement of other genes, as already suggested by other authors [16–22]. Broadening our analysis to a targeted resequencing approach led to the identification of pathogenic variants in *IFT140*, and variants of uncertain significance in *COL4A4, COL4A3, PKHD1, LRP5* and *ALG8*. Despite this further analysis, a specific genetic diagnosis was still missing for three patients. It is possible that other genes, still unidentified may be involved. According to several studies, ADPKD shows high genetic complexity, thus attributing the correct diagnosis and providing adequate genetic counseling to affected patients and their families is mandatory [23, 24]. This concept clearly emerged from our study, as the extension of the genetic analyses to non-*PKD1*/*PKD2* genes confirmed the diagnosis in five cases (41.7%), and described variants of uncertain significance in three cases (25.0%). Recent reports [25] described *IFT140* in ADPKD-like phenotypes, including both kidney and liver cysts, and suggesting a link in > 1% of cases. The percentage of negative-*PKD1*/*PKD2* patients in our cohort with a variant in *IFT140* (41.7%) was higher than that reported in the literature (20%), even though the disease was generally mild, as expected.

Regarding the other mutated genes, as reported in the literature, the ones related to Alport syndrome (*COL4A3*, *COL4A4*) can be associated with kidney cysts. Gulati and colleagues observed that collagen IV gene mutations were present in a series of patients with bilateral kidney cysts, suggesting that type IV collagen mutations may be present in ADPKD patients without mutations in the *PKD1* or *PKD2* genes [16].With regard to *PKHD1*, although the perinatal manifestation is the most common form, later onset was also described in the literature [23]. *ALG8* was another gene involved in the development of kidney and liver cysts, even though the severity of the disease was mild [22].

Regarding kidney function, progression has been reported as slower in these patients [3–6]. Determining the number of kidney cysts and the volume growth patterns may provide additional insights into the clinical management and treatment in ADPKD patients, as the growth in kidney volume directly stems from the increase in kidney cyst volume [26]. As described by Irazabal et al. [3], the only significant predictors for kidney function progression in Class 2 ADPKD patients are eGFR at baseline and atrophic vs non atrophic radiological presentation. In the literature, height-adjusted total kidney volume was observed to be the least predictive tool for Class 2 patients [5]. Neither height-adjusted total kidney volume nor the slope of total kidney volume resulted significant [26, 27], as opposed to Class 1 patients. We evaluated kidney function trend in our cohort, confirming the presence of mild kidney disease in our patients (only two subjects presented CKD stage III or higher, with a mean age of 54.4 ± 13 years). There were only two rapid progressors, according to the definition provided in the 2022 ERKNet Position Statement [28]. The absence of a trend towards severe progression, albeit in agreement with previous literature data, could also be related to the limited number of patients in our cohort. When assessing eGFR in Class 2A and Class 2B patients, our analysis did not find a significant difference in the eGFR (*p* value = 0.9) or in the eGFR slope (*p* value = 0.1).

In this study, we also aimed to evaluate total cyst volume. As already observed by Cadnapaphornchai and colleagues, total cyst volume assessment could be a useful tool in children with ADPKD [29]. Furthermore, in a longitudinal study conducted by Bae et al*.* [30], the authors used total cyst volume to describe the growth of cysts and kidneys over time. Despite using a different method to measure total cyst volume, our findings did not reveal any significant differences in either total cyst volume or residual tissue volume between patients with preserved or impaired kidney function, but a difference between Class 2A and 2B is highlighted (*p* value = 0.04). The significant increase in residual tissue volume/total cyst volume and residual tissue volume/total kidney volume and the significant reduction in total cyst volume/total kidney volume in Class 2B may imply  the presence of a structural difference between the two classes of ADPKD, and warrant further validation in larger cohorts to link the structural aspect to functional behavior.

Extrarenal involvement and hypertension were present in a high percentage of cases in our cohort. In comparison, only Iliuta et al. [13] evaluated the presence of liver cysts, that were present in 87.0% of cases. This suggests the need to investigate the presence of extrarenal involvement in affected patients and in their family members, to better characterize the patients’ phenotype. Hypertension was present in more than 50% of cases, in line with other reports in typical ADPKD patients.

### Conclusions

In conclusion, Class 2 ADPKD patients present genetic and clinical differences compared to Class 1 subjects. Two patients carried a likely pathogenic mutation and a variant of uncertain significance in the *PKD1* gene. The research of a cystic phenotype in family members of affected subjects revealed significant cystic involvement with a more variable functional progression, thus suggesting the need for family screening. The decline in kidney function was observed to be slower. Extrarenal involvement and hypertension are frequent and are important issues that need to be assessed in affected patients.

Finally, residual tissue volume did not correlate with kidney function in Class 2 ADPKD patients. This result highlights that other imaging tools  should be investigated in future research in order to understand kidney prognosis in Class 2 patients.

## Data Availability

All data generated or analysed during this study are included in this published article.
